# Understanding the Relation Between Self-Compassion and Suicide Risk Among Adolescents in a Post-disaster Context: Mediating Roles of Gratitude and Posttraumatic Stress Disorder

**DOI:** 10.3389/fpsyg.2020.01541

**Published:** 2020-07-16

**Authors:** Aiyi Liu, Wenchao Wang, Xinchun Wu

**Affiliations:** Beijing Key Laboratory of Applied Experimental Psychology, National Demonstration Center for Experimental Psychology Education, Faculty of Psychology, Beijing Normal University, Beijing, China

**Keywords:** self-compassion, gratitude, PTSD, suicide risk, adolescents

## Abstract

**Background:**

The suicide risk among adolescents post-earthquake remains an important issue in trauma psychology. While existing studies and theories suggest that factors such as self-compassion, gratitude, and posttraumatic stress disorder (PTSD) play roles in the risk of suicide, few studies have combined these factors to explore the relationship between them.

**Objective:**

This study examined the mediating roles of gratitude and PTSD in the relationship between self-compassion and suicide risk among Chinese adolescents after the Ya’an earthquake.

**Methods:**

Four and a half years after the Ya’an earthquake, 499 middle school students in Lushan County were assessed using the following systems: Measures of Self-Compassion Scale, Gratitude Questionnaire, PTSD Checklist for DSM-5, and Child Behavior Problems Questionnaire.

**Results:**

When we controlled for gender, age, and traumatic exposure, in the direct effect model, positive self-compassion had a negative effect on suicide risk, and negative self-compassion had a positive effect on suicide risk. In the indirect effects model, both positive self-compassion and negative self-compassion had no significant direct effect on suicide risk. Moreover, we found an indirect and negative effect of positive self-compassion on suicide risk via gratitude and PTSD, as well as via an indirect path from gratitude to PTSD. On the other hand, we also found an indirect and positive effect of negative self-compassion on suicide risk via gratitude and PTSD, as well as via an indirect path from gratitude to PTSD.

**Conclusion:**

Positive self-compassion reduces the risk of suicide, while negative self-compassion increases the risk of suicide. Gratitude and PTSD play significant mediating role between self-compassion and suicide risk.

## Introduction

Earthquake is a major traumatic disaster that causes huge property loss, death, and injuries to people and results in various psychological problems among victims (Fan et al., 2011). Suicide is a terminal outcome in the spectrum of potential major mental health issues spawned by severe earthquakes that can hinder victims’ recovery to a normal life and further threaten people’s lives (Mezuk et al., 2009). Previous empirical studies have also found a significant increase in suicide risk among survivors after earthquakes (Tang et al., 2010). Importantly, adolescents are sensitive to traumatic events and likely produce negative psychological outcomes (Good and Willoughby, 2008). Therefore, paying attention to the suicide risk of adolescents after earthquakes is important, and identifying malleable protective and risk factors is necessary to improve the development of targeted interventions to reduce suicide risk.

### Self-Compassion Affects Suicide Risk

Previous research has focused on suicide risk in environmental, emotional, and cognitive factors (Dieserud et al., 2001; Esposito et al., 2003). According to the integrated motivational-volitional model of suicidal behaviors (O’Connor, 2011), self-attitude is an important influencing factor for the formation of suicidal ideation and development of suicidal behavior of individuals who have experienced negative events. However, research on suicide risk from the perspective of self-attitude remains lacking. Self-compassion, as a self-attitude, likely plays a role in the motivational phase of suicidal behavior among adolescents after earthquakes. Self-compassion means being kind and forgiving of oneself in the face of suffering, neither judgmental nor indifferent, and recognizing their suffering as a universal experience that all people can experience without feeling isolated and hopeless (Neff, 2003a). Self-compassion consists of six components (Neff, 2003b): *Self-kindness*, being positive and accepting of oneself; *common humanity*, seeing own suffering as something that can happen to anyone; *mindfulness*, looking at the situation with balance and clarity; *over-identification*, focusing on own negative emotions and flaws; *self-judgment*, being indifferent and rigorous of oneself; *isolation*, feeling lonely and helpless by making oneself as the center of suffering.

In previous studies, most scholars regard the six dimensions of self-compassion as a single structural variable, but other scholars divide self-compassion into positive and negative self-compassion; and this two-dimensional structure has been verified in certain studies (López et al., 2015; Montero-Marín et al., 2016). In general, self-kindness, common humanity, and mindfulness represent adaptive facets of self-compassion, that is, positive self-compassion; whereas self-judgment, isolation, and over-identification represent maladaptive facets of self-compassion, that is, negative self-compassion (Neff, 2003b). Certain studies have shown that positive and negative self-compassion has different effects on individual psychological outcomes (Montero-Marín et al., 2016). Specifically, positive self-compassion has a positive effect on positive psychological outcomes, whereas negative self-compassion has a positive effect on negative psychological outcomes (Gilbert et al., 2011; López et al., 2015; Muris and Petrocchi, 2017). Therefore, the present study also divides self-compassion into positive and negative self-compassion.

From the relationship between positive self-compassion and suicide risk, positive self-compassion may protect adolescents from engaging in suicide. Conceptually, positive self-compassion can be considered a positive self-attitude (Neff, 2003b). When individuals face negative events and overwhelming sufferings, positive self-compassion makes individuals care about their own feelings in a tolerant and gentle way and reduce negative thoughts about themselves (Raes, 2010; Krieger et al., 2013), thus reducing suicide risk. Moreover, positive self-compassion may help individuals obtains a clear and balanced mind and stay focused on the present without ruminating over and being stuck in overwhelming negative emotions (Selby et al., 2013; Heath et al., 2016). Chang et al. (2017) found that positive self-compassion can reduce suicide risk among American college students. In further exploring the underlying mechanisms by which positive self-compassion affects suicidal behavior, Rabon et al. (2019) argued that positive self-compassion can ultimately reduce suicide risk by alleviating individuals’ depression and anger.

Conversely, negative self-compassion may increase individuals’ suicide risk. The differential activation theory of suicidality (Lau et al., 2004) suggests that suicidal behavior is one of the most globally representative, negative self-related thought patterns. When negative emotions are activated by negative events related to individuals, such negative emotions further trigger individuals’ hopelessness and powerlessness, thus activating suicidal ideation and ultimately increasing suicide risk. Negative self-compassion, as a negative factor related to the self, can make individuals immersed in painful emotions, which they cannot escape from and thus exacerbate their depression (Joeng and Turner, 2015). Individuals who are troubled by painful emotions can experience self-criticism, increase hopelessness and helplessness (Rogers et al., 2017), and ultimately increase suicide risk. Previous empirical studies have also demonstrated the relationship between negative self-compassion and suicide risk (Bonner and Rich, 1987; O’Connor, 2007; Joiner et al., 2009).

Although many theoretical and empirical studies have shown that self-compassion affects suicide risk, the predictive mechanisms of self-compassion in relation to suicide risk remain unclear. As a common post-traumatic negative psychological outcome, post-traumatic stress disorder (PTSD) is ubiquitous among post-traumatic adolescents and has a significant impact on their mental health (Zhou et al., 2017).

### Mediating Role of PTSD

In terms of the relationship between self-compassion and PTSD, the risk factor model proposed by Freedy et al. (1992) has the most extensive influence. This model suggests that self-attitude, as a pre-disaster factor, may be a predisposition factor leading to individuals’ psychosomatic response after trauma, which may have an impact on PTSD. Specifically, individuals with high levels of positive self-compassion tend to be caring and understanding to themselves in suffering and treat the frustration and guilt of traumatic events with a tolerant attitude, thus quickly adapting to the effects of trauma and ultimately helping alleviate PTSD symptoms (Barnard and Curry, 2011). Empirical studies have also shown that among children who experience large fires, children with positive self-compassion have low levels of PTSD symptoms (Zeller et al., 2015). Hiraoka et al. (2015) found that positive self-compassion alleviates PTSD symptoms among Iraqi soldiers. By contrast, a positive relationship exists between negative self-compassion and PTSD. Cognitive model points out that individuals who pay too much attention to traumatic experiences and potential threats can form negative emotions and cognition, which may aggravate PTSD (Ehlers and Clark, 2000). Negative self-compassion, on the other hand, causes individuals to excessively focus on trauma cues, keeps them immersed in painful emotions, and forms negative cognition, which may aggravate PTSD symptoms (Dunmore et al., 2001). At the same time, individuals with negative self-compassion tend to feel guilt and have self-criticism after traumatic events. Self-criticism and associated guilt may be the central maintaining and motivating factors in many PTSD cases (Ehlers and Clark, 2000; Mayou et al., 2002; Cox et al., 2004; Wang et al., 2020).

Previous studies have found that an increase in the number and severity of PTSD symptoms is associated with suicidal risk, including ideation, attempts, and death by suicide (Freeman et al., 2000; Panagioti et al., 2011; Wang et al., 2018). The interpersonal theory of suicide (Joiner et al., 2009) suggests that the ability to commit suicide is determined by prior painful and traumatic experiences, which, in turn, result in habituation to the fear and pain of death. The habit of suffering and lack of fear of death are important factors leading to individual suicide. As a complex, heterogeneous disorder, PTSD can immerse people in constant emotional experiences of pain and fear and relive these traumatic experiences repeatedly, which makes people less afraid of death and likely commit suicide (Panagioti et al., 2009). Previous studies of survivors after traumatic events have also found a link between PTSD and suicide risk. Caldera et al. (2001) investigated survivors of hurricane disasters and found that individuals with high levels of PTSD have a significantly increased risk of suicide. Tarrier and Gregg (2004) observed patients with chronic PTSD symptoms and revealed that the higher the level of PTSD, the greater the risk of suicide. This finding is consistent with the results of other studies revealing a high level of suicide risk in clinical samples with PTSD (Amir et al., 1999; Kotler et al., 2001). Therefore, PTSD possibly mediates the relationship between self-compassion and suicide risk.

### Mediating Role of Gratitude

After natural disasters, governments’ vigorous construction and interpersonal support and help likely arouse victims’ gratitude; thus, gratitude is a common positive emotional factor after traumatic events (McCullough et al., 2004). Gratitude can be defined as “a generalized tendency to recognize and respond with grateful emotion to the role of other people’s benevolence in the positive experiences and outcomes that one obtains” (McCullough et al., 2002). According to McCullough et al. (2002), the gratitude is positively associated with positive emotions, life satisfaction, vitality, and optimism, and negatively associated with depression and stress. Further, grateful people have a positive attitude toward themselves and others and they generally think that life is meaningful, understandable, and manageable (Lambert et al., 2009). Certain researchers believe that positive emotional factors play roles in the influence of self-compassion on the negative psychological outcomes of post-trauma victims (Arimitsu and Hofmann, 2015). Therefore, gratitude may be another mediator between self-compassion and suicide risk.

So far, only few studies have directly focused on the relationship between self-compassion and gratitude. However, the correlation between both variables can be inferred on the basis of relevant theories and research results. According to emotional experience theory, a clear perception of the benefits and help of others is the decisive factor to generate gratitude (Lazarus and Lazarus, 1996). Positive self-compassion emphasizes a clear and balanced way for individuals to perceive their current emotions as they experience suffering, rather than over-amplify pain and sadness (Neff, 2003a). Thus, individuals with high levels of positive self-compassion are speculated to clearly perceive gratitude when they receive favor and help. Furthermore, Neff (2003a) argued that individuals with positive self-compassion can treat themselves with tolerance and kindness. When people treat themselves with tolerance, they also accept the kindness and help shown by others with an open and tolerant attitude, which likely stimulates gratitude. Rao and Kemper (2017) tested participants’ scores for related positive emotions after training in a course to promote positive self-compassion. They found that individuals trained in positive self-compassion have significantly improved their gratitude scores. Rao and Kemper (2017) aimed to cultivate participants’ ability of self-compassion in a course. The researcher measured participants’ scores for related positive emotions after the course and found that individuals trained in positive self-compassion show significant increase in gratitude.

However, negative self-compassion may negatively affect gratitude. According to the disengagement hypothesis, when individuals are psychologically exposed to stress, excessive attention to traumatic cues can occupy their several cognitive resources that can make them focus on the negative aspects of traumatic events; experiencing positive emotions also becomes difficult for these adolescents (Koster et al., 2011). However, as a self-attitude that immerses individuals in painful emotions after trauma, negative self-compassion can magnify negative emotion and cognition, thus occupying several cognitive resources, which may be unconducive to the full feeling of gratitude when receiving help from others. Neff et al. (2007) observed that self-criticism triggered by negative self-compassion is significantly and negatively correlated with gratitude. Caputo (2015) also found a significant negative correlation between isolation and gratitude. On the basis of these findings, we can speculate that negative self-compassion may hinder the development of gratitude in adolescents after earthquakes.

The broaden-build theory of positive emotions (Fredrickson, 2004) suggests that gratitude can expand the cognitive schema and behavior pattern and provide sustained psychological resources for individuals (Folkman and Moskowitz, 2000). Consequently, individual happiness increases, and interpersonal relationship improves, which can promote growth and development in life (McCullough et al., 2002). Given that suicide risk is an aspect of negative outcomes in adolescent development (Portes et al., 2002), we tentatively predict that adolescents with high levels of gratitude have low risk of suicide. This hypothesis has been supported in previous empirical studies. Li et al. (2012) investigated the suicide risk of 1,252 Chinese middle school students from Guangzhou and found that gratitude can significantly reduce the suicide risk of teenagers. Similarly, Rey et al. (2019) focused on school bullying among high school students and explored the causes of suicidal behavior among adolescents in the context of trauma. They found that gratitude is an important protective factor for suicidal behavior in adolescents.

Posttraumatic stress disorder and gratitude are two potential mediators of the relationship between self-compassion and suicide risk. A combined relationship also exists between them. Numerous studies have shown that gratitude is an important positive emotion that can effectively suppress PTSD (Fredrickson et al., 2003; Kashdan et al., 2006; Israel-Cohen et al., 2014). Research with adults overwhelmingly indicates that gratitude is strongly related to healthy psychological and social functioning because it focuses people on self-improvement and helps them maintain and build strong, supportive social ties (Emmons and McCullough, 2003). After a traumatic event, access to supportive resources is a critical factor in an individual’s recovery from PTSD symptoms. Kashdan et al. (2006) focused on Vietnam veterans and revealed that participants with PTSD have significantly lower levels of gratitude than those without PTSD; the experience of gratitude also help veterans with PTSD recover daily functioning. Zheng et al. (2011) conducted a survey among 1,439 students who experienced the Wenchuan earthquake. The results showed that gratitude has a significant negative effect on PTSD.

### Present Study

In summary, although the relationship between the three variables of self-compassion, namely, gratitude, PTSD, and suicide risk have been examined in previous studies, the roles of gratitude and PTSD in the impact of self-compassion on suicide risk have not been explored. In addition, previous studies have found that self-compassion is a protective factor for suicide, but most have not classified the types of self-compassion (López et al., 2015; Montero-Marín et al., 2016). Therefore, the present study explores the effects of positive and negative self-compassion on suicide risk in adolescent survivors and examines the mediating roles of gratitude and PTSD.

## Materials and Methods

### Participants and Procedures

Four and a half years after the Ya’an earthquake, we selected two middle schools in Lushan County, Sichuan Province for investigation. Before conducting the investigation, we fully communicated with the local education bureau, informed relevant departments of our research content and methods, and obtained the approval.

With the assistance of the school leaders and head teachers of the two middle schools, we selected first and second grades of both junior high school and senior high school to conduct the investigation. Within these grades, we issued a total of 520 questionnaires, of which 21 questionnaires were screened out due to excessive missing values or failure to submit on time. A total of 499 valid questionnaires were recovered. All of the students who participated in the survey took a collective test during their classes on the same day. The mean age of the adolescents at the time of measurement was 14.94 (standard deviation = 1.58) years, and the range was from 12.0 to 20.0 years. Of the 499 students, 230 (46.1%) were male, and 264 (52.9%) were female; five participants did not report their gender. All participants had experienced the earthquake.

This study was approved by the Research Ethics Committee of the Beijing Normal University and conducted with the permission of the relevant leaders of the participating schools. All the participants signed a written informed consent. Considering that all our participants were juveniles under the age of 18, a written informed consent was also obtained from their parents before conducting the survey.

In this study, graduate students majoring in psychological counseling were given a collective test with uniform instructions. In the guidance of the questionnaire, we emphasized that the purpose of this survey is to explore the impact of the Jiuzhaigou earthquake experience on the participants’ current psychological responses. Before filling out the questionnaire, ask the participants to carefully read the guideline of the topic and fill out the questionnaire as required. All questionnaires were immediately recalled after completion.

### Measurements

#### Traumatic Exposure Questionnaire

The traumatic exposure questionnaire developed by Wu et al. (2013) was used in this study. The questionnaire consists of 18 items. The questions involved whether the participants had either witnessed or heard of others injured or killed in the earthquake. Each item was rated on a three-point Likert scale, ranging from 1 (*did not experience the situation above*) to 3 (*saw it myself*). Finally, the scores of all participants were added up as indicators of the degree of traumatic exposure. The internal reliability of the questionnaire in this study was acceptable (Cronbach’s α = 0.65).

#### Self-Compassion Scale

Participants were given the 26-item Self-Compassion Scale (Neff, 2003b), which has six components and is divided into positive and negative self-compassion. Positive self-compassion includes common humanity, self-kindness, and mindfulness. Negative self-compassion includes isolation, self-judgment, and over-identification. All the items were rated on a five-point Likert scale ranging from 1 (*completely disagree*) to 5 (*completely agree*). In this study, the internal consistency of positive self-compassion (Cronbach’s α = 0.96) and negative self-compassion (α = 0.96) was good.

#### Gratitude Questionnaire

We adopted the gratitude scale compiled by McCullough et al. (2002) and revised by Zhou and Wu (2017). This questionnaire consists of six items, among which the third and sixth items are reverse scoring questions. Each item was rated on a seven-point Likert scale ranging from 0 (*completely disagree*) to 6 (*completely agree*). In this study, the Chinese-revised gratitude questionnaire had good internal consistency (Cronbach’s α = 0.81).

#### PTSD Checklist for DSM-5

The PTSD checklist for DSM-5 was designed by Weathers (2013) and is a well-known instrument to assess post-traumatic stress symptoms. This checklist consists of 20 items and four subscales: intrusions, negative cognition and emotion alteration, avoidance, and hyper-arousal. Each item was rated on a four-point Likert scale ranging from 0 (*completely disagree*) to 3 (*completely agree*). The internal reliability of the questionnaire in this study was good (Cronbach’s α = 0.93).

#### Child Behavior Problem Questionnaire

The Youth Risk Behavior Survey Questionnaire prepared by Brener et al. (2004) was used to assess the suicide risk of adolescents. This questionnaire contains 19 items, and we adopted one of the subscales incorporating three items to measure suicide risk. The three items assessed participants’ suicidal ideation, plans, and attempts over the past six months. Each item was rated on a three-point Likert scale ranging from 1 (*not at all*) to 3 (*always*). The Chinese version of the questionnaire has achieved good reliability and validity among Chinese adolescents after earthquakes (Ying et al., 2015; Zhou and Wu, 2017), and the internal consistency reliability in this study was acceptable (Cronbach’s α = 0.84).

#### Data Analysis Strategies

We adopted the self-report method. All data used were continuous variables, and full-information maximum likelihood estimates were used to fill in missing data. All analyses were performed using software SPSS 24.0 and Amos 17.0. In the structural equation modeling (SEM), we used the following indicators to evaluate the model fitting: chi-square test of model fit (χ^2^/*df*), comparative fit index (CFI), Tucker–Lewis index (TLI), and root mean square error of approximation (RMSEA).

We then established SEM to examine the following models: (a) a direct effect model with structural paths from positive and negative self-compassion to suicide risk; (b) an indirect effect model with mediators (e.g., gratitude and PTSD) inserted between self-compassion (e.g., positive and negative self-compassion) and suicide. Given that gender, age, and trauma exposure may be important additional influencing factors in this study, we controlled for these variables in investigating the effects of self-compassion on suicide risk and the underlying mechanisms.

## Results

### Descriptive Statistics and Correlations

The results of the description statistics and correlation analysis of main variables are shown in Table 1. According to Pearson’s correlation analysis, gender was significantly associated with negative self-compassion, gratitude, and PTSD. However, the correlation with other variables was insignificant. Age was associated only with PTSD but not with other variables. No significant correlation was found between traumatic exposure and other variables and between positive self-compassion and PTSD. The correlation among other main variables was significant.

**TABLE 1:** Means, standard deviations, and correlations among the main variables.

	Mean	SD	1	2	3	4	5	6	7	8
1 Gender^a^	–	–	–							
2 Age	14.94	1.58	–0.03	–						
3 Traumatic exposure	20.81	4.31	–0.03	–0.02	–					
4 PSC	39.23	8.52	0.08	0.00	0.01	–				
5 NSC	37.74	8.84	0.16***	0.07	–0.01	0.50***	–			
6 Gratitude	24.39	6.92	0.13**	0.04	0.06	0.33***	0.10*	–		
7 PTSD	13.51	9.96	0.12*	0.11*	–0.06	–0.03	0.35***	−0.17***	–	
8 Suicide risk	3.67	1.31	0.03	–0.01	–0.04	−0.10*	0.19***	−0.27***	0.46***	–

Coded ^*a*^: 1, male; 2, female; PSC, positive self-compassion; NSC, negative self-compassion; PTSD, posttraumatic stress disorder. *p < 0.05; **p < 0.01; ***p < 0.001.

### SEM Analyses

We used SEM to examine the mediating effects of gratitude and PTSD between self-compassion and suicide risk. First, we estimated the measurement model, which included three latent variable constructs: positive self-compassion, negative self-compassion, and PTSD. We found that the model fitting index was acceptable [χ^2^/*df* = 3.82, CFI = 0.97, TLI = 0.96, RMSEA (90% CI) = 0.075 (0.059–0.088)]. Further structural model analysis is also possible.

**FIGURE 1:**
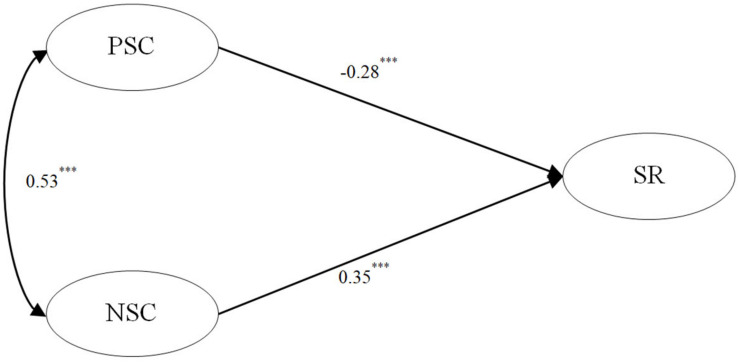
The direct effects model after controlling gender, age, and traumatic exposure. PSC, positive self-compassion; NSC, negative self-compassion; SR, suicide risk. ^∗∗∗^*p* < 0.001.

Second, after controlling for the effects of gender, age, and traumatic exposure, we established a direct effect model and added two direct effect paths from positive/negative self-compassion to suicide risk (Figure 1). We found that the data fitting of the direct effect model was good [χ^2^/*df* = 3.65, CFI = 0.96, TLI = 0.94, RMSEA (90% CI) = 0.073 (0.059–0.087)]. In the path analysis results, positive self-compassion had a negative effect on suicide risk (β = −0.28, *p* < 0.001), and negative self-compassion had a positive effect on suicide risk (β = 0.35, *p* < 0.001).

**FIGURE 2:**
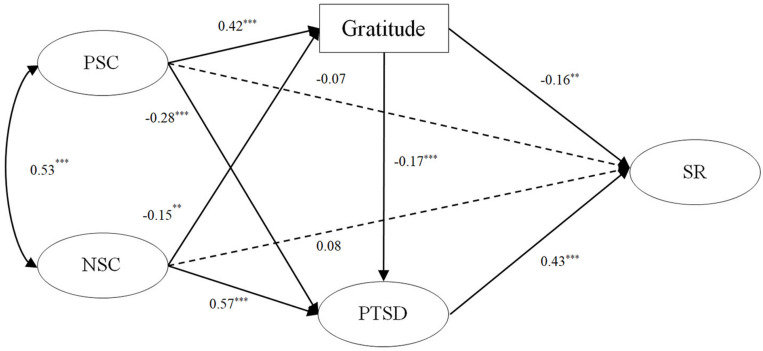
The multiple indirect effects model after controlling gender, age, and traumatic exposure. PSC, positive self-compassion; NSC, negative self-compassion; PTSD, posttraumatic stress disorder; SR, suicide risk. ^∗∗^*p* < 0.01; ^∗∗∗^*p* < 0.001.

Third, based on the direct effect model, we added two mediating variables, namely, gratitude and PTSD between self-compassion and suicide risk. Moreover, based on previous theories and studies (Fredrickson, 2001; Fredrickson et al., 2003; Kashdan et al., 2006), we added a path from gratitude to PTSD and structured a multiple indirect effect model (Figure 2). The results of the model revealed that the fitting index was acceptable [χ^2^/*df* = 2.87, CFI = 0.96, TLI = 0.95, RMSEA (90% CI) = 0.061 (0.052–0.071)]. In the path analysis results, positive self-compassion had a significant positive effect on gratitude (β = 0.42, *p* < 0.001) and had a negative effect on PTSD (β = −0.28, *p* < 0.001), whereas negative self-compassion had a significant negative effect on gratitude (β = −0.15, *p* < 0.01) and had a positive effect on PTSD (β = 0.57, *p* < 0.001). Gratitude had a significant negative effect on suicide risk (β = −0.16, *p* < 0.01), while PTSD had a positive effect on suicide risk (β = 0.43, *p* < 0.001), and gratitude had a significant negative predictive effect on PTSD (β = −0.17, *p* < 0.001).

To further verify the significance of the mediating effects, the bias-corrected bootstrap method was used to calculate the mediating effect values and their 95% CI. Table 2 illustrates these results, which showed an indirect and negative effect of positive self-compassion on suicide risk via gratitude and PTSD (β = −0.07, 95% CI = −0.12 to −0.03; β = −0.12, 95% CI = −0.17 to −0.06) and via an indirect path from gratitude to PTSD (β = −0.03, 95% CI = −0.03 to −0.02). Negative self-compassion had a positive effect on suicide risk via gratitude and PTSD (β = 0.02, 95% CI = 0.01 to 0.04; β = 0.25, 95% CI = 0.18 to 0.30) and via an indirect path from gratitude to PTSD (β = 0.01, 95% CI = 0.00 to 0.11). These results suggested that gratitude and PTSD play complete mediating roles between self-compassion and suicide risk.

**TABLE 2:** Bias-corrected bootstrap tests of mediating effects.

Path	Standardized β	Standardized 95% CI	
		Low	High
PSC-Gratitude-SR	−0.07*	–0.12	–0.03
NSC-Gratitude-SR	0.02*	0.01	0.04
PSC-PTSD-SR	−0.12**	–0.17	–0.06
NSC-PTSD-SR	0.25***	0.18	0.30
PSC-Gratitude-PTSD-SR	−0.03*	–0.03	–0.02
NSC-Gratitude-PTSD-SR	0.01*	0.00	0.01

PSC, positive self-compassion; NSC, negative self-compassion; SR, suicide risk; PTSD, posttraumatic stress disorder. *p < 0.05; **p < 0.01; ***p < 0.001.

## Discussion

After controlling for the effects of gender, age, and trauma exposure in the direct effect model, we found that the positive self-compassion of post-disaster adolescents has a direct negative effect on suicide risk, which supports previous theories and studies (Chang et al., 2017; Rabon et al., 2019). This result indicates that positive self-compassion can help individuals gain a clear and balanced mind and stay focused on the emotions of the moment, rather than ruminate and immerse in overwhelming negative emotions (Selby et al., 2013; Heath et al., 2016), which can ultimately reduce adolescents’ suicide risk.

In the indirect effect model, we observed that negative self-compassion has a direct and positive role in the suicide risk among adolescents. The results support the differential activation theory of suicidality (Lau et al., 2004), which is consistent with previous research (Joeng and Turner, 2015). Negative self-compassion activates self-related negative emotions, such as self-criticism, guilt, and shame (López et al., 2015), which make individuals feel hopeless and powerless. When adolescents are immersed in painful emotions for a long time and unable to deal with difficult situations, they likely resort to suicidal behaviors to escape from the current pain (Brown et al., 2002).

Importantly, we found that in the indirect effect model, positive self-compassion reduces the suicide risk among adolescents through the mediating effect of gratitude, which verifies emotional experience theory (Lazarus and Lazarus, 1996) and the broaden-build theory of positive emotions (Fredrickson, 2004). For adolescents who have experienced earthquakes, the high level of self-compassion allows them to deal with the suffering in a calm and balanced attitude, rather than being plagued by pain and despair (Thompson and Waltz, 2008). This state allows adolescents to clearly perceive gratitude. Furthermore, high levels of gratitude can expand adolescents’ cognitive and behavioral pattern; reduce negative cognition, emotion, and behavior (Fredrickson, 2004); and increase adolescents’ life satisfaction and happiness (McCullough et al., 2002). These factors can reduce the psychological impact of traumatic events on adolescents and ultimately reduce the risk of suicide.

The current study also suggests that negative self-compassion exerts an indirect and positive effect on suicide risk via gratitude, which is consistent with theories and studies (Fredrickson et al., 2003; Kashdan et al., 2006; Neff et al., 2007; Koster et al., 2011; Caputo, 2015). Adolescents with negative self-compassion tend to see themselves as the center of pain after a disaster and focus too much cognitive resources on the negative cues of the traumatic event (Neff, 2003a). Therefore, adolescents cannot fully feel gratitude after receiving favors from others. Adolescents with low levels of gratitude are also at increased risk of suicide because they have been troubled by negative emotions and cognition for a long time.

Consistent with previous studies (Leary et al., 2007; Neff et al., 2007; Vettese et al., 2011; Zeller et al., 2015), positive self-compassion has a negative effect on suicide risk through PTSD. Specifically, adolescents with positive self-compassion tend to view traumatic events as disasters that are possible for all. Thus, viewing the impact of a disaster on the self with an objective and peaceful mind and caring for and treating the self with kindness after the trauma is easy to avoid thinking over the negative aspect of the traumatic event and finally alleviate the symptoms of PTSD. As one of the most important predictors of suicide risk (Joiner et al., 2009) in adolescents who experience negative events, a decrease in PTSD levels can correspondingly reduce the risk of suicide.

In addition, we observed that negative self-compassion has an indirect and positive effect on suicide risk via PTSD. Negative self-compassion in post-traumatic adolescents can exacerbate PTSD symptoms, and these findings confirm previous studies (Dunmore et al., 2001; Mayou et al., 2002). According to cognitive model (Ehlers and Clark, 2000), adolescents with negative self-compassion can magnify the sense of helplessness and despair they experience in the face of traumatic events and regard themselves as the center of pain, thereby focusing cognitive resources on negative emotions and ultimately aggravating PTSD, which can increase adolescents’ suicide risk. On the basis of the interpersonal theory of suicide (Joiner, 2005), adaptation to pain and lack of fear of death are important factors that increase the risk of suicide. Adolescents with PTSD continuously and repeatedly experience painful emotions and fear of death from major life-threatening traumatic events. This habituation process causes them to lose their fear of death and become prone to suicidal ideation and behavior in the face of overwhelming pain to get rid of the pain (Panagioti et al., 2009).

Moreover, we found that self-compassion has a two-mediator indirect effect on suicide risk through gratitude via PTSD. Individuals with positive self-compassion experience high levels of gratitude; according to the broaden-build theory of positive emotions (Fredrickson, 2004), gratitude can promote positive emotions, help individuals expand their cognitive schemas (Folkman and Moskowitz, 2000), enhance behavioral flexibility (Fredrickson, 2004), construct personal resources (Fredrickson et al., 2003), and eliminate the physiological effects of negative emotions (Tugade and Fredrickson, 2004). These positive effects can reduce PTSD symptoms and, in turn, reduce suicide risk in adolescents who experience traumatic events. By contrast, adolescents with negative self-compassion have low levels of gratitude, which keeps PTSD from improving. This finding increases suicide risk for adolescents who are chronically troubled by PTSD.

However, we observed that the direct effect path from positive and negative self-compassion to suicide risk is insignificant. One possible explanation is that the mediating roles of gratitude and PTSD may cover the direct path of self-compassion and suicide risk.

## Conclusion

In summary, this study explored the effects of the two components of self-compassion on suicide risk in adolescents after an earthquake and the underlying mechanisms between them. The results showed that positive self-compassion reduces suicide risk, whereas negative self-compassion increases suicide risk. Gratitude and PTSD play significant mediating roles between self-compassion and suicide risk.

This result enriches the current research on post-traumatic adolescent mental health and provides advice and enlightenment to psychological intervention workers in clinical disaster areas. First, this study confirms the important effects of self-compassion on post-traumatic adolescents, and positive and negative self-compassion has different effects on suicide risk. The results suggest that clinical psychologists can introduce techniques related to self-compassion, such as Compassion-Focused Therapy, Mindful Self-Compassion, and Loving-Kindness Meditation (Barnard and Curry, 2011), when treating adolescents that are at risk of suicide. By means of the above psychological interventions, people can develop positive self-compassion and transform negative self-compassion caused by traumatic events, thus allow adolescents to reduce their negative emotions in the face of disasters, thereby reducing their risk of suicide.

Second, this study has demonstrated that gratitude is an important mechanism between self-compassion and suicide risk. However, previous research has shown that individual gratitude levels are relatively low during adolescence, because they have a limited span in terms of number of life circumstances for which they can feel grateful (Froh et al., 2011; Langher et al., 2016). Therefore, clinical psychologists are focusing on how to improve the gratitude of post-traumatic adolescents in order to reduce their suicide risk. The gratitude journal is one of the most widely used tools for practicing gratitude (Emmons and McCullough, 2003). In the process of psychological intervention for adolescents who have experienced a traumatic event, psychologists in school could incorporate this technique appropriately to promote an individual’s level of gratitude, thereby reducing their potential suicide risk.

Finally, psychological intervention workers in disaster areas should pay attention to the identification and diagnosis of survivors’ PTSD symptoms and timely give psychological treatment to reduce the risk of suicide.

Certain limitations exist in the study design and measurement. First, the research only controlled for gender, age, and trauma exposure but did not control for additional factors that may affect suicide risk. Considering that the psychological responses of individuals who have experienced traumatic events may be affected by other covariates, including and controlling for more covariates in future studies will diminish interference in the results. Second, the study was cross-sectional, which makes inferring causal relationships among variables difficult; future research must focus on this issue by using a longitudinal design. Third, the self-report method was uniformly adopted in this study, inevitably leading to a certain degree of common method biases. Future research should incorporate multiple methods to improve depth and quality of data. At last, all the variables in this study are self-reported by the subjects and have strong subjectivity, especially the measurement of gratitude, which is easily affected by the social desirability bias (Caputo, 2017). In future studies, individual’s gratitude should be measured by different test methods, so as to reduce the social desirability bias as much as possible.

## Data Availability Statement

The raw data supporting the conclusions of this article will be made available by the authors, without undue reservation, to any qualified researcher.

## Ethics Statement

This study was approved by the Research Ethics Committee of the Beijing Normal University and conducted with the permission of the relevant leaders of the participating schools. All the participants signed a written informed consent. Considering that all our participants were juveniles under the age of 18, a written informed consent was also obtained from their parents before conducting the survey.

## Author Contributions

AL developed the study design, participated in and supervised the data collection, performed the statistical analysis, and drafted the manuscript. WW participated in and supervised the data collection, assisted in data collection and analysis, and made important modifications to the manuscript. XW conceived the study and revised the manuscript critically for important intellectual content. All authors contributed to the article and approved the submitted version.

## Conflict of Interest

The authors declare that the research was conducted in the absence of any commercial or financial relationships that could be construed as a potential conflict of interest.
